# The Importance of Networking: Plant Polycomb Repressive Complex 2 and Its Interactors

**DOI:** 10.3390/epigenomes6010008

**Published:** 2022-03-03

**Authors:** James Godwin, Sara Farrona

**Affiliations:** Plant and AgriBiosciences Research Centre, Ryan Institute, NUI Galway, H91 TK33 Galway, Ireland; sara.farrona@nuigalway.ie

**Keywords:** PRC2, chromatin, protein interactors, H3K27me3, Arabidopsis, transcription, development

## Abstract

Polycomb Repressive Complex 2 (PRC2) is arguably the best-known plant complex of the Polycomb Group (PcG) pathway, formed by a group of proteins that epigenetically represses gene expression. PRC2-mediated deposition of H3K27me3 has amply been studied in Arabidopsis and, more recently, data from other plant model species has also been published, allowing for an increasing knowledge of PRC2 activities and target genes. How PRC2 molecular functions are regulated and how PRC2 is recruited to discrete chromatin regions are questions that have brought more attention in recent years. A mechanism to modulate PRC2-mediated activity is through its interaction with other protein partners or accessory proteins. Current evidence for PRC2 interactors has demonstrated the complexity of its protein network and how far we are from fully understanding the impact of these interactions on the activities of PRC2 core subunits and on the formation of new PRC2 versions. This review presents a list of PRC2 interactors, emphasizing their mechanistic action upon PRC2 functions and their effects on transcriptional regulation.

## 1. Background

Polycomb Repressive Complex 2 (PRC2) mediates the deposition of the trimethylation of the lysine 27 of the histone 3 (H3K27me3), a histone modification associated with gene repression in eukaryotes [1]. PRC2 was first identified in *Drosophila* consisting of four core components: Enhancer of zeste (E(z)), a histone methyltransferase unit that catalyses H3K27me3; Extra sex combs (Esc), a WD40 domain protein scaffolding the interactions within the complex; Suppressor of zeste 12 (Su(z)12), a Zinc Finger protein that is essential for binding to nucleosomes; and Nuclear remodeling factor (Nurf55, also called p55), a Trp-Asp (WD) repeat protein involved in nucleosome remodelling [1,2]. After discovering PRC2 complexes in *Drosophila* as regulators of *Hox* genes expression, homologs of PRC2 subunits were identified in plants and other organisms [3,4,5]. In *Arabidopsis thaliana* (*Arabidopsis*), there are three E(z) homologs—CURLY LEAF (CLF), SWINGER (SWN) and MEDEA (MEA); three Su(z)12 homologs—EMBRYONIC FLOWER 2 (EMF2), VERNALIZATION 2 (VRN2) and FERTILIZATION-INDEPENDENT SEED 2 (FIS2); a single Esc homolog—FERTILIZATION-INDEPENDENT ENDOSPERM (FIE); and there are five *Arabidopsis* homologs of p55 protein—MULTICOPY SUPPRESSOR OF IRA (MSI) 1–5, but MSI1 is the only one demonstrated to be part of the PRC2 complex [6,7]. Based on their different subunit compositions, at least three PRC2-like complexes controlling different developmental processes have been described in *Arabidopsis*: the EMF, VRN and FIS complexes [3].

In plants and animals, loss-of-function of core PRC2 subunits results in the abrogation of H3K27me3 levels in PRC2 target genes, which leads to serious developmental defects, highlighting the critical role of PRC2 in development [7,8]. In *Arabidopsis* chromatin, PRC2 components mimic H3K27me3 localisation [9]. Genome-wide profiling revealed that 20–25% of *Arabidopsis* genes were marked by H3K27me3, and these genes globally display low expression levels [10,11,12]. Similar percentages of H3K27me3 marked genes were observed in different plant model species (e.g., maize, oilseed rape, rice and *Brachypodium distachyon*) [13,14,15,16]. These data further demonstrate the importance of PRC2 activity in regulating the expression of key developmental genes in crops and thereby governing the major agricultural traits, e.g., flowering. Besides PRC2’s pivotal function in controlling development, its key role in the regulation of stress responses and other essential cellular processes, such as metabolism, is emerging [17,18,19], although still relatively less understood in both plants and animals. Furthermore, the cells perceive the dynamic environmental signals and translate it into differential chromatin and transcriptional states and this is mediated through histone reader proteins that bind to H3K27me3 and/or that affect local chromatin compaction [18,20]. In a quest to identify protein reader complexes, two plant-specific H3K27me3 readers, namely EARLY BOLTING IN SHORT DAYS (EBS) and its homolog SHORT LIFE (SHL), were recently discovered and were proposed to act within the PcG pathway causing gene repression [21,22]. However, a key question remains unanswered, how do these proteins coordinate their activities with PRC2 to regulate gene expression?

Epigenetic marks such as H3K27me3 can be stably inherited during somatic cell divisions but can be reset during major developmental phase transitions such as the formation of gametes and embryos [23]. In plants, unique mechanisms exist for the inheritance of H3K27me3 marks compared to their animal counterparts. For instance, a recent study in *Arabidopsis* demonstrated a global reduction in H3K27me3 in the paternal germline (i.e., sperm cells), achieved by the coordinated action of three mechanisms: (1) lack of expression of PRC2 histone methyltransferases encoding units such as *CLF*, *MEA* and *SWN*; (2) active removal by Jumonji-C family methylation erasers (histone demethylases); and (3) the global deposition of a sperm cell specific histone H3 variant, H3.10/HTR10, which is resistant to K27 methylation [24]. Overall, several mechanisms are being elucidated for the transgenerational memory of H3K27me3 in *Arabidopsis*, but there is much more yet to be discovered. 

**Figure 1:**
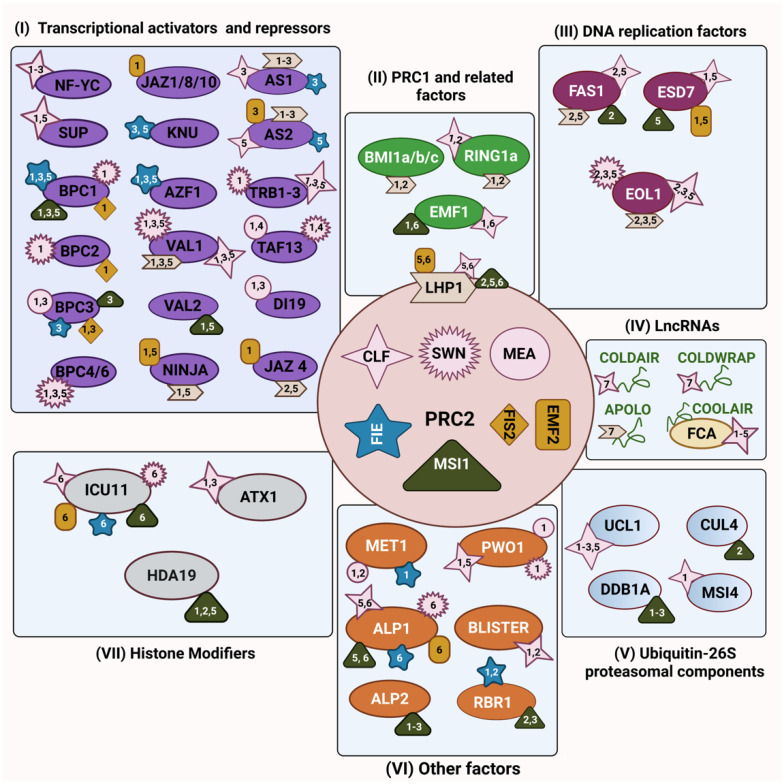
The physical interaction map of the PRC2 complex in *Arabidopsis*. Each of the PRC2 core components are represented in different shapes enclosed by a circular box in the centre, E(z) homologs are shown in pink colour—CURLY LEAF (CLF) as a four-pointed star, SWINGER (SWN) as a multi-pointed star and MEDEA (MEA) as a circle; Su(z)12 homologs are shown in golden yellow—EMBRYONIC FLOWER 2 (EMF2) as a rectangle, and FERTILIZATION-INDEPENDENT SEED 2 (FIS2) as a rhombus; the ESC homolog—FERTILIZATION-INDEPENDENT ENDOSPERM (FIE)—is represented as a blue five-pointed star; and the p55 protein homolog—MULTICOPY SUPPRESSOR OF IRA 1 (MSI1)—is represented as a dark green triangle. Physical interactors of PRC2 were functionally grouped into six categories: (**I**) transcriptional activators and repressors (purple); (**II**) PRC1 and related factors (light green); (**III**) DNA replication factors (magenta); (**IV**) long non-coding RNAs (green thread-like structure); (**V**) ubiquitin-26S proteasomal components (cyan blue); (**VI**) other factors (orange); and (**VII**) histone modifiers (grey). Physical interactors from each category may bind to one or more PRC2 components and the numbers (1–6) within the PRC2 component represent different confirmation techniques used for protein–protein interaction studies: 1—yeast two hybrid; 2—pull down assay; 3—biomolecular fluorescence complementation; 4—fluorescence resonance energy transfer; 5—co-immunoprecipitation; 6—co-immunoprecipitation coupled to mass spectrometry; 7—RNA-immunoprecipitation and binding assays. In the figure, LIKE HETEROCHROMATIN PROTEIN 1 (LHP1) is placed at the interface between PRC2 and PRC1. Figure created with BioRender.com (accessed on 21 January 2022).

In animals, the catalytic and non-catalytic function of PRC2 can be regulated by interaction with protein partners [25,26,27]. Similarly, plant PRC2 core components are associated with several other proteins, including PRC1 subunits, transcription factors, chromatin-related proteins, the replication machinery, and proteasomal components leading to the modulation of PRC2 activity and/or resulting in its recruitment to target genes [28]. Essentially, the physical interaction between PRC2 subunits and other proteins helps us to understand the intricate network of protein–protein interactions that occur to regulate PRC2-mediated gene repression during plant developmental transitions and in response to environmental signals. This review highlights the protein interactors of the *Arabidopsis* PRC2 core subunits identified so far (Figure 1). Nevertheless, VRN2 and its related VRN-PRC2 complex play a highly specialised role in vernalization-induced flowering that has already been extensively reviewed [29,30,31]; hence, we excluded its interactors. We discuss PRC2 recruitment strategies on target genes mediated by the cooperation with accessory proteins and its associated gene repression and explore the impact of PRC2 interactions especially on the modulation of PRC2 activities.

## 2. PRC2’s Interaction with Transcriptional Activators and Repressors

PRC2 recruitment to specific target genes has been elusive and we are just starting to understand how it occurs. One of the recruitment strategies of PRC2 is through intermediary DNA-binding proteins, which are able to bind specific DNA sequences to facilitate PRC2 recruitment to the chromatin [32,33,34]. DNA-binding transcription factors (TFs) and transcription-associated factors, both transcriptional activators and repressors, interact with PRC2’s components to target it to specific genes [35,36,37] (Figure 1).

Earlier studies in *Drosophila* revealed that TFs belonging to different families bind to *cis*-regulatory DNA elements of several hundred base pairs in length called Polycomb Response Elements (PREs). PREs are found in the promoter regions and are able to recruit PRC2 to target loci [38,39,40]. *Drosophila* PREs share little sequence homology, which has made its identification challenging in other multicellular organisms. Moreover, many of these sequence-specific DNA-binding factors of *Drosophila* lack clear mammalian orthologues and there is limited direct evidence to support sequence-specific PRC2 targeting in mammals despite several hundred equally highly conserved target genes between *Drosophila* and mammals [35,41]. However, a representative feature of *Drosophila* PREs is that they are enriched with binding motifs for several DNA-binding factors [42]. Interestingly, analyses of PRC2-target genes in *Arabidopsis* have revealed several *cis*-elements with PRE-like properties [36] with similar roles in PRC2 recruitment, which will be referred as plant PREs.

Plant PRE-based PRC2 recruitment mechanism was first demonstrated in *Arabidopsis* where MYB TFs, namely ASYMMETRIC LEAVES 1 (AS1) and AS2, form a complex and physically interact with PRC2 components (CLF, EMF2 and FIE) [43]. The AS1/AS2 complex was proposed to bind plant PREs in the Class I KNOX homeobox genes *BREVIPEDICELLUS (BP)* and *KNOTTED-ARABIDOPSIS THALIANA 2* (*KNAT2),* thereby recruiting PRC2 and resulting in stable H3K27me3-mediated gene silencing in differentiating leaves [44]. Therefore, an AS1/AS2 TF complex may act as a mediator for PRC2 recruitment and binding to specific PRC2 target genes.

Large scale yeast two-hybrid (Y2H) screening revealed that among several plant PRE-interacting TFs belonging to the Cys2-His2 (C2H2) zinc-finger (ZnF), the plant-specific APETALA2-like (AP2) and the BASIC PENTACYSTEINE (BPC) families physically interact with at least one PRC2 component [36]. Specifically, ARABIDOPSIS ZINC FINGER 1 (AZF1) and BPC1 interact with FIE, which is accompanied by overlapping chromatin occupancy of AZF1, BPC1 and FIE and with H3K27me3 peaks, both globally and at individual loci such as *AGAMOUS (AG)* and *SHOOT MERISTEMLESS (STM)* [36]. Interestingly, knockdown mutants of the *BPC* and *ZnF* TF families triggered upward leaf curling and precocious flowering, similarly to the phenotypes of *clf* mutants, with significant reduction in PRC2 (i.e., FIE) occupancy and H3K27me3 levels at several PcG target loci. BPC1 interacts and recruits FIE in vivo and triggers PRC2-mediated gene silencing in germinating seeds. In addition, it was demonstrated that AZF1 and BPC1 work together in PRC2 recruitment [36]. Another report showed that BPC4 interacts with SWN in bimolecular fluorescence complementation (BiFC) and co-immunoprecipitation (co-IP) assays, and further Y2H analyses revealed that BPC1, BPC2 and BPC6 specifically interact with SWN [45]. Further studies revealed that BPCs recruit PRC2 to the *ABSCISIC ACID INSENSITIVE 4* (*ABI4*) locus via a PRE-based mechanism and repress *ABI4* expression by increasing H3K27me3 levels in its promoter [45]. Similarly, class I BPCs, BPC1–3, interact with all four core FIS-PRC2 components in planta and bind to the *FUSCA3 (FUS3*) locus, thereby inducing the spatiotemporal repression of *FUS3* expression in developing seeds [46]. Furthermore, Class I and II BPCs act redundantly to repress the expression of the ovule identity gene *SEEDSTICK* (*STK)*, mediating the establishment of H3K27me3 marks via the PRC2 complex [47]. Taken together, these results demonstrate an intricated interplay between the members of the BPC family and PRC2.

The telomeric DNA-binding proteins TELOMERE-REPEAT-BINDING FACTORS 1–3 (TRB1–3), members of the Single-Myb-Histone protein family [48], directly interact with CLF and SWN [37]. The transcriptomic profile observed in the *trb1/2/3* triple mutant is similar to severe PRC2 mutant plants (i.e., the *clf;swn* double mutant), accompanied by a substantial redistribution of H3K27me3 levels [37]. In addition, TRB1 Chromatin Immunoprecipitation followed by sequencing (ChIP–seq) revealed a significant overlap with FIE and H3K27me3. TRB proteins bind to specific DNA elements known as telobox and related motifs recruiting PRC2 for H3K27me3 deposition at target genes [37]. Therefore, the molecular functions of TRB proteins indicate that they are not exclusively telomeric and indeed seem to play a more general role in chromatin remodelling via PRC2 that still needs to be fully elucidated.

TATA BINDING PROTEIN (TBP)-Associated Factor 13 (TAF13), a transcriptional regulator involved in seed development, interacts with MEA and SWN [49]. The loss of function of *TAF3* causes embryo arrest at the pre-globular stage, seed defects, and over-proliferation of endosperm similar to the mutant phenotype of the components of FIS-PRC2 [49]. Moreover, *taf13* mutants showed mis-regulation of the FIS-PRC2 seed developmental target genes *PHERES1 (PHE1), FUS3*, and *FORMIN HOMOLOGY5 (FH5)*, suggesting a possible role of TAF13 in the PRC2-mediated regulation of gene expression [49].

C2H2-type ZnF TF KNUCKLES (KNU) represses the homeobox gene *WUSHEL (WUS)* in the floral meristem, which is a target of PRC2-mediated repression [50]. A recent study from the same group demonstrated that KNU physically interacts with FIE and recruits PRC2 to the *WUS* promoter region to repress it via H3K27me3 accumulation. Hence, KNU acts as an integrator of PRC2-mediated transcriptional repression in the floral meristem [51].

*SUPERMAN (SUP)*, a flower-specific gene controlling the boundary of the stamen and carpel whorls, encodes a TF with a C2H2-type ZnF motif involved in floral organogenesis and floral meristem determinacy by fine-tuning auxin signalling [52]. It was known that the direct targets of SUP *YUCCA 1* and *4 (YUC1/4)*, involved in auxin biosynthesis, were regulated by deposition of H3K27me3 by PRC2 [11,52]. It was recently confirmed that direct SUP–CLF interaction may mediate the recruitment of PRC2 to at least some of its target genes, including *YUC1/4,* and coupling floral organogenesis and floral meristem determinacy [52].

DROUGHT-INDUCED 19 (DI19), another C2H2-type ZnF TF implicated in multiple abiotic stress signalling pathways [53], interacts with MEA. Furthermore, DI19 recruits MEA to the *RESISTANCE TO P. SYRINGAE* 2 (*RPS2*) promoter, resulting in increased H3K27me3 levels at the *RPS2* locus and subsequent decreased *RPS2* expression levels [54], showing one of the very few direct examples of the impact of PRC2 beyond development as a regulator of plant responses to biotic stress in *Arabidopsis.*

JASMONATE-ZIM DOMAIN (JAZ) proteins are transcriptional repressors involved in the perception of Jasmonyl-Isoleucine (JA-Ile), the active form of Jasmonic Acid (JA) that facilitates the transcriptional reprogramming of JA-responsive genes in response to developmental and environmental signals [55]. Y2H assays revealed that full-length *Arabidopsis* JAZ proteins including JAZ1, JAZ4, JAZ8 and JAZ10 directly interact with EMF2. Furthermore, pull-down and co-IP analysis revealed that JAZ4 interacts with EMF2. The accessory PRC2 component LIKE HETEROCHROMATIN PROTEIN 1 (LHP1), as we will further discuss, also interacts with seven out of the 13 known JAZ proteins [56]. Another transcriptional repressor, NOVEL INTERACTOR OF JAZ (NINJA), is an adaptor protein that is able to directly bind to most JAZs, and to EMF2 and LHP1 [56]. Thus, this recent report uncovered the concerted action and interaction of JAZ proteins with PRC2 factors to mediate transcriptional repression at various JA-responsive genes in *Arabidopsis*.

Nuclear transcription factor Y subunit C-1 (NF-YC1), also called as Histone-Associated Protein 5A (HAP5A), binds the CCAAT box, a frequently found *cis*-element in eukaryotic promoters [57]. NF-YC1 temporally interacts with CLF during floral transition [58]. The physical interaction of NF-YC1 and CLF antagonizes the association of CLF with chromatin and the CLF-dependent deposition of H3K27me3, subsequently allowing the expression of the florigen-encoding gene *FLOWERING LOCUS T* (*FT*) and inducing flowering under long-day conditions [58]. How the NF-YC1-CLF interaction exactly interferes with CLF binding to the chromatin at molecular level is an interesting question that still requires further investigation.

VIVIPAROUS1/ABI3-LIKE1 (VAL1) and VAL2 are DNA-binding B3 domain proteins that recognize the six-nucleotide RY motif and are essential for the transition from embryonic to vegetative growth by engaging PRC2 for the silencing of embryonic genes [59,60,61,62]. More recently, Chen et al. (2020) confirmed that VAL1 and VAL2, which are able to form homo and heterodimers, repress the *DELAY OF GERMINATION 1* (*DOG1*) gene, which encodes one of the most significant seed dormancy regulators, primarily through the PRC2-mediated deposition of H3K27me3 at this locus. VAL1 interacts with MSI1 and, furthermore, the interaction of VAL1 and VAL2 with SWN, CLF and LHP1 was confirmed by BiFC, co-IP and/or Y2H assays [59,63,64]. Previously, it had been observed that the phenotype of *val1;val2* mutant seedlings partly resembled the strong *swn;clf* double mutant [65]. In addition, the *val1;val2* double mutant showed a reduction in H3K27me3 deposition at the *FLOWERING LOCUS C* (*FLC*) locus [33] and genome-wide profiling revealed spatial redistribution of H3K27me3 in the *val1;val2* double mutant, which strongly affected transcription [64]. Besides the physical interaction of VAL1/2 and PRC2 components, genome-wide studies revealed that *val1;val2* significantly reduces SWN and CLF enrichment at PRC2 target loci [64]. Despite their role in PRC2-mediated repression, it is worth noting that a significant proportion of VAL1- and VAL2-occupied regions were not associated with SWN and CLF or H3K27me3, indicating further roles of VAL1 and VAL2 in genetic regulation beyond their PRC2-related activities [64].

In summary, the recruitment of PRC2 on target genes seems to depend on the coordinated action of DNA-binding proteins/TFs that can recognize different types of plant PREs. Moreover, another possibility is that different TFs may have affinity towards the same plant PRE, adding an extra layer of complexity as some TFs may have antagonistic, mutualistic or synergistic roles in their binding to plant PREs. In animals, it was demonstrated that multiple interactions of proteins can occur at PREs: (1) among diverse DNA-binding factors; (2) between DNA-binding factors and PcG members, including those of PRC2; and (3) between different PRC2s [66]. In plants, the elucidation of unknown DNA-binding factors/TFs, the identification and conservation of novel plant PREs, and the understanding of how they mechanistically cooperate with PRC2 subunits will be key to further decipher the mechanisms of PRC2 recruitment to target genes.

## 3. Interaction between PRC2 and PRC1 Components

In contrast to PRC2, PRC1 is highly divergent between plants and animals and several plant-specific proteins have been identified as PRC1 components [5,67,68]. The PRC1 complex harbours E3 ligase activity for catalysis of H2A monoubiquitination (H2Aub) [69]. In *Arabidopsis*, PRC1 was proposed to consist of the conserved subunits B LYMPHOMA Mo-MLV INSERTION REGION ONE HOMOLOG 1a/1b/1c (AtBMI1a/1b/1c) and the REALLY INTERESTING NEW GENE 1a/1b (AtRING1a/1b) [67,70]. The plant-specific proteins LHP1 and EMF1 have been initially proposed to be PRC1 components [71,72,73,74]. However, this hypothesis is still debatable and will be discussed in this section.

In *Arabidopsis*, the chromodomain-containing protein LHP1, also known as TERMINAL FLOWER 2 (TFL2), physically associates with PRC1 components such as AtRING1a, AtBMI1a, AtBMI1b and AtBMI1c in Y2H and pull-down assays [72,75] and also co-purifies with PRC2 components in co-immunoprecipitation coupled with mass spectrometry (coIP-MS) experiments [76]. Specifically, LHP1 co-purifies with CLF and EMF2 [77] and direct interaction of LHP1 with MSI1 was proposed to trigger a positive feedback loop to establish full H3K27me3 levels at target genes [77]. LHP1 homologs have been identified in many other plant species [78,79,80,81], but interestingly, LHP1 binds to H3K27me3 marks in vitro and associates with genes marked by H3K27me3 in vivo [10,82], while its best known *Drosophila* ortholog, HETEROCHROMATIN PROTEIN 1α (HP1α), recognises and binds to the heterochromatic mark H3K9me2 but not the H3K27me3 mark [80,82,83]. Another report revealed a physical association of LHP1 with PRC2 interactors, AS1 and AS2, which subsequently mediates PRC2 recruitment on *BP* and *KNAT2* loci to establish H3K27me3 [84]. In *Arabidopsis*, *lhp1* mutant seedlings showed no global changes of H3K27me3 distribution, suggesting that LHP1 does not have an overall role in the deposition of the H3K27me3 mark [82]. However, further studies on specific PRC2 target genes, including *FLC* and *SEPALLATA* 3 (*SEP3*), demonstrated that H3K27me3 levels are significantly decreased in *lhp1* [77]. In addition, similar altered genome-wide spreading of H3K27me3 in the gene bodies was observed in *lhp1* and *clf* mutants [85]. The transcriptional profiles of *clf* and *lhp1* mutants are significantly correlated, suggesting that both proteins may be involved in related pathways [72,77,86]. Therefore, taken together, the protein–protein interaction and epigenetic profiles indicate that LHP1 may act as an interface subunit between the PRC2 and PRC1 complexes (Figure 1), as it interacts with members of both complexes and affects the enrichment of the H3K27me3 mark in at least a set of PRC2 target genes [77,85,87].

The plant-specific EMF1 promotes vegetative growth and represses flowering [88]. Loss-of-function mutants in the *EMF1* gene mimic the pleiotropic phenotype of the *fie* and *mea* mutant [89]. The plant-specific EMF1 was initially thought to be a PRC1 component as it interacts with AtBMI1a and AtBMI1b in in vitro pull down assays [72] and mediates chromatin compaction in vitro similarly to the activity of the *Drosophila* PRC1 component Posterior sex combs (Psc) [71,73]. Nevertheless, EMF1 is required for H3K27me3 marking at PcG target genes [71,90] but not required for H2Aub activity as this second histone mark seems unaltered genome-wide in *emf1* [84,85]. Moreover, the EMF1 binding pattern is similar to the pattern of H3K27me3 deposition [91]. *EMF1* and *EMF2* genetically interact with each other and participate in silencing of the floral homeotic genes *AG*, *PISTILLATA* (*PI*) and *APETALA3* (*AP3*) [91]. However, there is no experimental evidence to show any direct physical interaction between EMF1 and EMF2. Nevertheless, EMF1 copurifies with other PRC2 members [77,87,92]; in particular, EMF1’s interaction with MSI1 and CLF was confirmed by coIP-MS and in vitro pull down assays [71,87].

Overall, EMF1 and LHP1 not only interact with PRC1 subunits but also copurify with PRC2 components [71,77,87]. Therefore, it is possible that EMF1 and LHP1 may participate in two different and independent complexes: 1) a PRC1-like complex (AtRING1/AtBMI1/EMF1/LHP1) with H2Aub catalysing activity and 2) a PRC2/EMF1/LHP1 complex that is able to catalyse H3K27 trimethylation. A third possibility proposes the existence of a PRC1-like complex containing LHP1 that is able to interact with CLF-PRC2 via the MSI1 subunit, since LHP1 copurifies with MSI1 in co-IP [77]. Supporting this hypothesis, mutants affected on core *Arabidopsis* PRC1 components (i.e., the *ring1a/b* and *bmi1a/b* mutants) showed severe developmental phenotypes similar to the ones displayed on strong PRC2 mutants, such as *clf;swn, fie* or *emf2;vrn2,* suggesting a possible functional interaction between the two complexes [72,93].

Hence, the relationship between the PRC1 and PRC2 components is probably far more complicated than initially thought. Several studies have shown the physical interaction between PRC2 and PRC1 components, suggesting that their functions are closely integrated [74,75,77,87]. Contrasting pieces of evidence are also emerging on the interdependency of PRC2 and PRC1 for their activities. Initially, the canonical model postulated in animals to explain the hierarchy between both complexes was also proposed in plants, i.e., PRC1’s activity would depend on its ability to sequentially bind to PRC2-mediated H3K27me3 [94]. More recent studies demonstrated that different scenarios for the crosstalk between both complexes may exist as, at least for a set of PcG-regulated genes, H3K27me3 activity relies on PRC1 activity [93,95]. For example, in the repression of seed maturation genes during post-germination, H2Aub precedes H3K27me3, demonstrating that PRC1 can also work upstream of PRC2 [93,96]; however, it was only shown in a handful of target genes. The classic hierarchical model of PcG recruitment was definitely challenged by the recent genome-wide chromatin data, which demonstrated that PRC1 can act independently of PRC2 activity as a different set of genes was marked with only H2Aub or H3K27me3, also indicating that these marks may play independent roles [97,98]. However, it is not fully understood whether H2Aub or H3K27me3 marks depend on different recruiting factors or if these factors can function synergistically at target genes. Moreover, plant-specific PRC1 components are not well defined yet and an increasing number of PRC1 associated proteins, including VERNALIZATION 1 (VRN1), have just recently been identified in *Arabidopsis* [99,100]. Therefore, although current data envisage much more dynamic and versatile scenarios for the relationship of the two PRCs than the one depicted by the initial hierarchical model, further studies will be required to understand the underlying mechanisms of PRC2 and PRC1 recruitment and their dependent or independent activities on chromatin for the epigenetic regulation of gene expression. The biochemical purification and characterisation of plant PcG complexes in a cell-specific manner will become crucial to reveal in which chromatin context they carry out their functions.

## 4. PRC2’s Interaction with Ubiquitin-26S Proteasomal Components

In the last decade, there has been emerging evidence of the regulation of PRC2 components by their interaction with members of the ubiquitin-26S proteasome, especially with E3 ubiquitin ligases, which facilitate the transfer of ubiquitin to a substrate [93,94,95]. The ubiquitination of proteins may cause subsequent protein degradation [101], but can also promote changes in the function or activity of the ubiquitinated proteins including chromatin-associated proteins [102]. The ubiquitination of core PRC2 subunits for the control of PRC2 activity and the subsequent protein turnover of PRC2 components will be discussed here.

UPWARD CURLY LEAF1 (UCL1), a plant-specific F-box component of the well-characterised Skp, Cullin, F-box (SCF)-containing E3 ligase complex, physically associates with CLF in the nucleus and subsequently ubiquitinates CLF to target it for degradation via the ubiquitin-26S proteasome pathway. This interaction seems to be quite specific as UCL1 does not interact with MEA [103]. Overexpression of *UCL1* reduces CLF protein levels and alters the expression levels of CLF target genes, suggesting a negative regulation of CLF by UCL1 [103]. Moreover, the phenotypes of mutants affected in *UCL1* and *CLF* indicate that they may act in the same genetic pathway in which UCL1 may be a negative regulator of CLF [103].

Another multimeric E3 ubiquitin ligase complex contains CULLIN 4 (CUL4), a scaffolding protein, and DAMAGED DNA-BINDING PROTEIN 1 (DDB1), an adaptor protein that associates with the substrate protein and targets it for degradation [104]. In *Arabidopsis*, DDB1 physically interacts with MSI1 and CUL4, indicating the possibility for a CUL4–DDB1–MSI1 protein complex [105]. The question was asked as to whether MSI1 could act as a substrate receptor of this E3 ligase complex. The results from two independent studies revealed that MSI1 protein turnover is indeed not under the control of CUL4 [105,106]. However, when CUL4’s function was compromised, silencing of paternal *MEA* was released in the seeds due to the reduction in H3K27me3 levels at this locus and overall [105], pointing to a mediation of CUL4–DDB1 in the activity of the FIS–PRC2 complex. In the *cul4* mutant, there was significant decrease in H3K27me3 levels on *FLC* and its downstream target *FT* [106], further supporting CUL4-DDB1 function in the regulation of PRC2 activities.

CUL4 and DDB1 physically interact with another p55 ortholog, MSI4, and form the CUL4–DDB1–MSI4 complex. MSI4 also interacted with CLF, but not FIE, in Y2H and in planta BiFC assays [106]. Furthermore, loss-of-function mutations of *MSI4* reduce H3K27me3 on *FLC* and *FT*, resulting in their upregulation and causing a late-flowering phenotype. Therefore, direct regulation of CLF–PRC2 activity by the CUL4–DDB1–MSI4 E3 ubiquitin ligase was plausible [106]. Recently, a plant-specific protein, EMBRYO DEFECTIVE 1579 (EMB1579), implicated in embryo development [107], was demonstrated to recruit and phase condensate CUL4–DDB1–MSI4 [108]. In addition, EMB1579 facilitates the physical association of the CUL4–DDB1–MSI4 complex with CLF and contributes to maintaining the proper H3K27me3 levels on *FLC*, subsequently controlling flowering [108]. In animals, studies are emerging on how ubiquitination modulates liquid–liquid phase separation of PRCs to mediate large-scale chromatin compaction [109], whereas in plants, there is increasing evidence of the conservation and importance of liquid–liquid phase separation in the organisation of the nuclear space [110]. Understanding the interface among ubiquitination, liquid–liquid phase separation and PRC2 may provide key mechanistic insights into PRC2’s recruitment and dynamics.

## 5. PRC2’s Interaction with DNA Replication Components

During cell division, PRC2’s interaction with DNA-replication-related proteins enables the transmission of H3K27me3 to the daughter cells. Understanding PRC2-mediated gene silencing in a replication-coupled manner through its interaction with members of the replication machinery is crucial to dissect the molecular mechanisms behind the inheritance of the H3K27me3 mark on canonical and histone variants in post-replicative chromatin. Thus, in this section, we will highlight the physical association of PRC2 with components of the DNA replication machinery (Figure 1).

### 5.1. FASCIATA 1

CHROMATIN ASSEMBLY FACTOR 1 (CAF-1) is an evolutionarily conserved heterotrimeric chaperone complex that facilitates the association and deposition of histone tetramers (H3 and H4) onto nascent chromatin [111,112]. In *Arabidopsis*, three subunits, namely FASCIATA1 (FAS1), FAS2 and MSI1, form the functional CAF1 complex in vitro [113]. The analyses of mutants affected in CAF-1 subunits revealed its essential role in controlling pollen development and apical meristem architecture [114,115]. FAS1’s direct interaction with CLF, LHP1 and AtRING1A was confirmed by in vitro pull-down and co-IP assays [115]. Strikingly, FAS1 colocalises with PRC2 and PRC1 components, within the DNA replication foci, suggesting that both PRC2–CAF1 and PRC1–CAF1 interactions occur at the DNA replication sites [115], to further illustrate the possible interplay between both PRCs. In addition, CAF1 deposits the histone variant H3.1 at the replication fork and facilitates the maintenance of H3K27me3 in the new synthesised DNA molecule [115].

### 5.2. ENHANCER OF LHP1 (EOL1)

ENHANCER OF LHP1 (EOL1) is a plant homolog of yeast Chromosome transmission fidelity 4 (Ctf4), which acts in the DNA helicase complex during DNA replication [116]. EOL1 is a nuclear protein produced in dividing cells and is associated with the replication machinery in *Arabidopsis* [117]. EOL1 physically interacts with SWN, CLF and LHP1. The *eol1* mutant acts as an enhancer of the *clf* mutant and *eol1;clf* plants have smaller rosette leaves and flower earlier than *clf* plants. H3K27me3 levels at *FT*, *AG* and *SEP3* were increased in *eol1;clf* but remained unchanged in the *eol1* single mutant. In addition, some H3K27me3-enriched genes showed increased expression in *eol1;lhp1* compared to *lhp1* mutants since the loss of function of EOL1 further increases the misexpression of H3K27me3 target genes that are already upregulated in the *lhp1* mutant. Overall, this study proposed that EOL1 function is required for LHP1-PRC2 to maintain H3K27me3 levels at target genes in dividing cells [117], as EOL1 is exclusively expressed in actively dividing cells and is required for the inheritance of H3K27me3 marks during replication.

### 5.3. DNA Polymerases

In *Arabidopsis, EARLY IN SHORT DAYS 7 (ESD7) (also called POL2a*/*ABA OVERLY SENSITIVE 4* (*ABO4*)) encodes the catalytic subunit of the DNA Polymerase epsilon (Pol ε), which is involved in the synthesis of the leading DNA strand during replication and has been found to be essential for the viability of the embryo [118,119]. ESD7 physically interacts with CLF, EMF2 and MSI1. CLF and EMF2 are recruited to *FT* and *SUPPRESSOR OF OVEREXPRESSION OF CO 1* (*SOC1)* chromatin by ESD7 to maintain the H3K27me3 levels on these loci [120]. Mutants of other DNA polymerases subunit-encoding genes, such as Pol-α *INCURVATA2* (*ICU2*) and Pol-δ *POLD2,* impact H3K27me3 distribution in several genes and enhance the abnormal phenotype of PcG mutants [121]; however, a direct interaction of these DNA polymerases with PRC2 subunits still needs to be demonstrated. Despite these promising links, the role of DNA polymerases in nucleosome reconstitution and the way in which the deposition of post-translational histone modifications is coupled to the activity of these enzymes remain elusive.

## 6. PRC2’s Interaction with Histone Modifiers

The functional implications of histone modifications for the recruitment of PRC2, or in the regulation of its activities, is not well understood. Importantly, co-occurring of histone modification appears to influence PRC2 activity and there is an intricated orchestration of PRC2 binding to other histone-modifying enzymes. In this section, we will explore the interaction of PRC2 with histone modifiers (Figure 1).

### 6.1. INCURVATA 11 (ICU11)

In *Arabidopsis*, *INCURVATA 11* (*ICU11)* encodes a 2-oxoglutarate-dependent dioxygenase (2OGD). The 2OGD domain of ICU11 belongs to the same enzymatic superfamily as Jumonji C-domain histone demethylases [92]. The *icu11* mutant shows a slight increase in H3K36me3 levels, an active histone mark, suggesting a role of ICU11 in H3K36me3 demethylation [92]. The *icu11* mutant shares many pleotropic phenotypes with the *emf1* and *emf2* mutants (e.g., small-sized cotyledon, leaf curling and early flowering) and ICU11 copurifies with CLF, SWN, FIE, MSI1 and EMF2 and other PRC2 accessory components such as EMF1, LHP1 and TRB1–3 [92]. The physical cooperation between histone methyltransferases and demethylases has been proposed to contribute to positive feedback loops for the transition between opposite chromatin stages (e.g., from open to closed chromatin conformation) and modelling studies in *Schizosaccharomyces pombe* predict that this kind of physical coupling facilitates the bi-stability of opposing chromatin states [122,123]. In *Arabidopsis*, another example that validates this hypothesis is the interaction of the H3K27me3 demethylase EARLY FLOWERING 6 (ELF6) with the H3K36me3 methyltransferase SET DOMAIN GROUP 8 (SDG8). In this case, the ELF6-SDG8 interaction switches chromatin from a closed to an open stage [124].

### 6.2. ARABIDOPSIS HOMOLOG OF TRITHORAX 1 (ATX1)

ARABIDOPSIS HOMOLOG OF TRITHORAX 1 (ATX1) catalyses the deposition of H3K4me3 and belongs to the Trithorax Group (TrxG) pathway, which plays an antagonistic role in PRC2 proteins by means of gene activation [125]. ATX1 and CLF physically bind to each other in Y2H and BiFC assays despite their, in principle, antagonistic activities. Loss-of-function mutations in *ATX1* or *CLF* genes result in the repression or activation of the floral homeotic gene *AG*, respectively [126]. Interestingly, a lack of both ATX1 and CLF functions results in partial restoration of H3K4me3 and H3K27me3 on the *AG* nucleosomes. On the other hand, restoring *AG* repression rescues the respective single-mutant phenotype (*atx1* and *clf*). Therefore, it is suggested that ATX1 and CLF-coordinated activities generate the bivalent marks H3K4me3 and H3K27me3 at the *AG* locus [126]. Besides the *AG* locus, these types of bivalent chromatin marks, mediated by TrxG and PcG, were reported at other loci such as *FLC*, *SUP* and *APETALA 1* (*AP1*) [17,126]. In animals, the presence of H3K4me3 and H3K27me3 marks at silent embryonic stem cell loci has been proposed to act as an inducer of a bivalent transcriptional state that reduces noise and that poses genes for transcription later in development [127]. However, more recent models have been suggested in yeast in which the antagonistic TrxG/PcG interplay is required for the bistable regulation of target genes [123].

### 6.3. HISTONE DEACETYLASES (HDAC)

HISTONE DEACETYLASES (HDACs) catalyse the deacetylation of lysine residues in histones and regulate gene expression [128]. To date, a few HDACs from plants have been characterised. In *Arabidopsis*, the most studied HDACs, HISTONE DEACETYLASE 6 (HDA6), HDA9 and HDA19, are involved in the regulation of developmental processes and environmental responses [129,130,131]. It is shown that HDA19 co-purifies with MSI1 to fine-tune ABA signalling by binding to ABA receptor genes [132]. Recently, HDA9, a homolog of HDA19, was shown to preferentially deacetylate H3K27 to pave PRC2-mediated H3K27me3 deposition at various loci, resulting in transcriptional repression. These findings also suggest that H3K27 deacetylation may be a prerequisite for H3K27me3 activity and gene repression [133,134]. Furthermore, it was demonstrated that HDA9 and HDA19 are required for PRC2 enrichment on *FLC* chromatin [134]. Another report showed that HDA9 and HDA19 physically associate with VAL1 and VAL2, and VAL2 was also reported to bind HDA6 [59,135], suggesting that some HDACs are interlinked with PRC2 through VAL proteins. Overall, it seems that there is a concerted action between at least certain HDACs and PRC2 for the repression of specific target genes in *Arabidopsis*, whether this is conserved throughout the HDAC family and in other plant species is still an unresolved question.

## 7. Other PRC2 Interactors

In this section, we would like to introduce other PRC2 interactors that cannot be easily categorised in one specific group but have proved to play a crucial role in the regulation of chromatin-related processes and gene expression through their interaction with PRC2 components (Figure 1).

### 7.1. RETINOBLASTOMA RELATED 1 (RBR1)

Retinoblastoma protein (pRb), a cell-cycle-regulatory element initially identified as a tumour suppressor in humans, regulates the progression from G1 to S phase [136]. RETINOBLASTOMA RELATED 1 (RBR1) is a plant orthologue of pRb, which was first demonstrated to act as a negative regulator of the cell cycle [137]. RBR1 is required for persistent repression of the late embryonic gene, *LEAFY COTYLEDON2* (*LEC2*), by increasing H3K27me3 levels via PRC2. Reduced RBR1 function in seedlings arrested development after germination, suggesting its crucial role in seedling establishment [138]. Earlier studies conducted to understand PRC2–RBR1 link found that FIE binds to the pRb orthologs of *Arabidopsis* and maize, confirmed by pull-down and Y2H assays, and the protein sequences of plant pRb orthologs that participate in this interaction have been well conserved throughout evolution [139]. In addition, loss of RBR1 activity also perturbs the expression of genes that encode PRC2 subunits, such as *FIS2*, *SWN* and *CLF* [140]. Reciprocally, PRC2-specific H3K27me3 activity represses the paternal *RBR* allele in the embryo and endosperm during seed development. Thus, these results revealed a functional repressive regulatory RBR1-PRC2 circuit involving cellular differentiation and reproductive development [140]. In addition, MSI1 interacts with RBR1 via the RbA domain of RBR1 in vivo, and together they directly down-regulate *METHYLTRANSFERASE 1 (MET1)* during female gametogenesis, thereby resulting in the transcriptional activation of the MET1 targets *FIS2* and *FWA* [141]. Therefore, RBR1’s cooperation with PRC2 is essential during reproductive development but the implications of this relationship in later developmental stages and its concerted activities upon other loci are still unknown.

### 7.2. DNA METHYLTRANSFERASE 1 (MET1)

In plants, MET1, also known as DECREASED DNA METHYLATION 2 (DDM2), maintains the DNA methylation of symmetric CpG residues [142]. MET1 physically interacts with MEA and FIE in the context of FIS-PRC2 [143]. Mammalian orthologs of MEA and MET1, i.e., EHZ2 and DNA METHYLTRANSFERASE 1 (DNMT1), respectively, were also reported to directly interact with each other [144]. Moreover, *MET1*, *MEA* and *FIE* share overlapping expression patterns in reproductive tissues during the early stages of development, which may obviously be necessary to allow their physical interaction of the proteins they encode [143]. Although the phenotypes observed in mutants affected in components of FIS-PRC2 are unrelated to the ones observed in *met1* single mutants [145], mutations in *MEA* act as enhancers of *met1* [143]. Notably, the synergistic effects of MEA and MET1 in the repression of endosperm development in the absence of fertilization were observed [143]. Therefore, these results indicate that the interplay between two of the major epigenetic pathways involved in histone and DNA methylation establishes or reinforces the silencing of common target genes during seed development. It will be very interesting to test if similar synergistic effects occur later during development.

### 7.3. PWWP-DOMAIN INTERACTOR OF POLYCOMBS 1 (PWO1)

PWWP DOMAIN INTERACTOR OF POLYCOMBS1 (PWO1) protein interacts with all three *Arabidopsis* PRC2 histone methyltransferases (i.e., CLF, SWN and MEA) and through its conserved PWWP domain, PWO1 can also bind to histone 3 (H3) [146]. In *Nicotiana benthamiana (N. benthamiana),* PWO1 changes the nuclear localisation of CLF and recruits CLF to subnuclear speckles, suggesting that PWO1 may act as a recruiter of PRC2 components to subnuclear domains [146]. In *Arabidopsis*, PWO1 does not homogeneously localises in the nucleus either [147]; however, how the PWO1 localisation pattern is determined, and which specific roles develop, are still unknown. *pwo1* mutants act as enhancers of *clf* and show decreased H3K27me3 enrichment at a subset of PRC2 targets in *pwo1* mutants. Nevertheless, these H3K27me3 changes were also accompanied by a reduction in H3 levels, hence indicating that PWO1 may be required for proper nucleosome occupancy to create an appropriated chromatin environment for the deposition of the PRC2-associated mark [146]. Another study identified PWO1 as a member of the PEAT (PWWPs–EPCRs–ARIDs–TRBs) complex, which is shown to be required for histone deacetylation and heterochromatin silencing [148]. In addition, PWO1 interacts with proteins associated with the nuclear periphery, such as CROWDED NUCLEI1 (CRWN1), which aid in the maintenance of nuclear morphology [147]. Strikingly, a small overlapping set of PRC2 target genes related to stress responses are upregulated in *pwo1* and *crwn1;crwn2* mutant plants, which may indicate a role of PWO1 in recruiting PRC2 and/or PRC2-regulated genes involved in the response to the environment [110]. Overall, PWO1 plays a crucial role in chromatin-associated gene repression at least partially through its interaction with PRC2. However, the exact molecular function of the PWO1–PRC2 partnership and the question of whether PWO1 could act as a link between PRC2 and other chromatin-related pathways will need to be addressed.

### 7.4. ANTAGONIST OF LIKE HETEROCHROMATIN PROTEIN 1 and 2 (ALP1 and 2)

Transposons usually encode in their sequence an enzymatic machinery as well as DNA components that have undergone co-option (i.e., a shift in the function of the trait) by the host genome via molecular domestication [149]. There are several examples of transposon-derived genes arisen by the evolutionary process of domestication. For instance, *ANTAGONIST OF LIKE HETEROCHROMATIN PROTEINS (ALPs)* are ancient, conserved plant-specific genes that belong to a distinctive *PIF*/*Harbinger* superfamily of transposons. *PIF/Harbinger* transposons encode two proteins, a protein with DNA-binding activity and a transposase with DNA endonuclease activity [150], whereas other transposon families carry out both activities by a single protein [151]. *Arabidopsis ALP1* likely lost its transposase activity as a process of molecular domestication and acquired a novel function during angiosperm evolution. In *Arabidopsis*, *ALP1* and *ALP2* were firstly identified in a genetic screen for suppressors of *lhp1* [87]. ALP1 and ALP2 physically interact with each other [152]. Further, coIP-MS experiments revealed that ALP1 associate with the core PRC2 components SWN, CLF, EMF2, FIE and MSI1, but not with the associated components LHP1 or EMF1 [87]. The association of ALP1 with CLF and MSI1 was confirmed by co-IP assays and BiFC [87]; Y2H and pull-down assays confirmed that ALP2 interacts with MSI1 [152]. Moreover, transient expression assays in tobacco showed that the interaction of ALP1 and MSI1 occurs in the presence of ALP2, further suggesting that ALP2 may act as a bridge for the ALP1-PRC2 association. Hence, the current working model is that ALPs-PRC2 is formed by the recruitment of ALP1 by ALP2 via MSI1 and that this complex lacks the accessory components, LHP1 and EMF1 [152]. In terms of developmental phenotype, *alp* mutants show a slight late-flowering phenotype, suggesting that ALPs are implicated in floral induction. Mutations in the *ALP* genes also act as suppressors of the *clf-28* mutation, and in *alp1/2;clf28* double mutants, H3K27me3 levels at the PRC2 target genes *SEP3*, *AG* and *FLC* were partially restored, reducing their mis-regulation. These results indicate that ALPs may counteract PRC2 activities. Thus far, these two reports on domesticated transposases that have acquired a novel function as PRC2 components open a new paradigm in plant epigenetics, proposing that the association of a domesticated transposase as an inhibitory component of PRC2 may have arisen because its beneficial role for the host [152]. Therefore, understanding the evolution of other domesticated Harbinger transposases and their associations with the chromatin-modifying machinery, such as PRC2, may unravel novel mechanisms involved in gene regulation and nuclear pathway evolution, providing novel molecular tools to manipulate plant chromatin.

### 7.5. BLISTER

BLISTER (BLI) is a plant-specific coiled-coil protein required for normal organ development, which controls cotyledon and leaf patterning by preventing premature cellular differentiation in *Arabidopsis*. As the name indicates, *bli* mutants display blister-like structures in several organs. BLI interacts with CLF in Y2H, pull down and split-luciferase assays in *N. benthamiana* and co-localisation of both proteins was observed in the nuclei of *N. benthamiana* [153]. A more recent article showed that, in addition to its nuclear localisation, BLI also localises in the Golgi [154]. Transcriptomic analysis of *bli-1* seedlings revealed a significant overlap of genes regulated by BLI and CLF. However, *bli* mutants do not show changes in H3K27me3 levels at analysed PRC2 target genes, suggesting it may act downstream or in parallel to the PRC2 pathway [155]. Previously, it had been demonstrated that BLI promotes resistance to cold stress in *Arabidopsis* [156] and, more recently, transcriptional up-regulation of several stress-responsive genes involved in endoplasmic reticulum stress, drought, high salt and heat stress was also observed in the *bli-1* mutant [155]. Overall, BLI performs a key role in plant development and stress-responses, although its molecular function is not well understood yet. Therefore, identification of BLI protein partners and analysis of its target genes during stress will improve our understanding of the molecular functions of BLI and its possible interplay with PRC2 in the transcriptional regulation of stress-responsive genes.

## 8. PRC2’s Interaction with Long Non-Coding RNAs

Another emerging paradigm is long non-coding RNAs (lncRNAs) controlling gene expression via structural and regulatory interactions with PRC2 [157,158]. Many PRC2-associated lncRNAs have been identified in mammals [159,160]. Similarly, in *Arabidopsis*, *COLD-ASSISTED INTRONIC NONCODING RNA (COLDAIR)* and *COLD OF WINTER-INDUCED NONCODING RNA FROM THE PROMOTER (COLDWRAP)* physically associate with CLF and target PRC2 to repress *FLC* [161,162]. Another intergenic lncRNA, *AUXIN-REGULATED PROMOTER LOOP (APOLO)*, interacts with the PRC2 accessory component LHP1 and modulates local chromatin 3D conformation [163]. Furthermore, *APOLO* participates in the *trans*-action mechanism of PcG recruitment through the formation of DNA–RNA duplexes (R-loops) and thereby controls the lateral root development in *Arabidopsis* [164]. However, the underlying biochemical principles are still unknown and how R-loops mediate PRC2’s targeting to chromatin remains to be elucidated.

FLOWERING CONTROL LOCUS A (FCA) is an RNA-binding protein that has a WW domain that is essential for protein–protein interaction and two RNA recognition motifs (RRM) [165]. In *Arabidopsis*, *fca* mutant plants show a late-flowering phenotype, whereas overexpression of *FCA* leads to early flowering under long-day and short-day conditions [166]. FCA interacts with CLF, which was confirmed by Y2H, pull down, BiFC and co-IP assays. FCA also directly binds COOLAIR, an *FLC* antisense transcript that plays a key role in repressing *FLC* transcription, and hence, may act as a functional link between COOLAIR and CLF. The FCA-COOLAIR-CLF interaction allows PRC2 to be targeted to *FLC* and increase H3K27me3 levels [167]. Loss of COOLAIR function results in a reduction in FCA binding to *FLC* and an enrichment of CLF at the same locus, which subsequently decreases H3K27me3 levels at *FLC* and induces its transcription [167]. In mammalian cells, RNA-binding proteins interact with PRC2 to mediate H3K27me3 deposition [168]. Therefore, understanding the nexus between regulatory lncRNAs, RNA-binding proteins and PRC2 will become key for a deeper understanding of complex gene-regulatory networks.

## 9. Conclusions and Perspectives

The last decade has seen a major advancement in our understanding of how PRC2 functions are regulated. The discovery of novel PRC2 partners has essentially contributed to this knowledge. Although PRC2’s main subunits are well conserved between plants and animals, the current landscape of PRC2 interactors in *Arabidopsis*, and the emerging one in other plant species, demonstrates that many of the accessory proteins that can directly bind PRC2 subunits are unique to plants. Throughout evolution, PRC2 may have recruited specific interactors in the plant cells to acquire novel and plant-specific activities that are key to the regulation of plant development and responses. Therefore, evolutionary studies to address why plants needed to invent these interactions, such as the ones currently carried out in ancestral plant species [169], will aid in clarifying PRC2’s phylogeny and elucidating its contribution to the evolution of plant development and adaptation [4]. The advancement in proteomic techniques, such as the affinity purification coupled to mass spectrometry (AP/MS) TAP assay [170], has proven to be crucial for revealing protein–protein interactions. More sophisticated techniques, such as, for instance, proteomic profiling of single cells [171], will be key for the further discovery of PRC2 partners and identification of plant-specific PRC2 versions.

Most of our knowledge of PRC2 and its interactors originated in *Arabidopsis*. More recently, genome-wide enrichment of H3K27me3 in important crops, such as rice, maize, barley and oilseed rape, has been made available [14,16,172], demonstrating a similar epigenomic landscape but also special features in the deposition of this mark. Phylogenetic analyses also demonstrate a good conservation of the proteins of the complex and the possible existence of similar PRC2 subcomplexes [4,173]. However, much more research is needed to understand how PRC2’s functions are regulated in these species through the conservation of its protein network or through the formation of novel species-specific interactions. We propose that a better understanding of PRC2 interactors in species of agronomic interest is capital to the discovery of new molecular tools for a tighter control of plant development and responses and for the breeding of new crop varieties with enhanced traits to better adapt their development to the environment.
